# Modulation of Glia Activation by TRPA1 Antagonism in Preclinical Models of Migraine

**DOI:** 10.3390/ijms232214085

**Published:** 2022-11-15

**Authors:** Chiara Demartini, Rosaria Greco, Giulia Magni, Anna Maria Zanaboni, Benedetta Riboldi, Miriam Francavilla, Cristina Nativi, Stefania Ceruti, Cristina Tassorelli

**Affiliations:** 1 Department of Brain and Behavioral Sciences, University of Pavia, Via Bassi 21, 27100 Pavia, Italy; 2 Unit of Translational Neurovascular Research, IRCCS Mondino Foundation, Via Mondino 2, 27100 Pavia, Italy; 3 Department of Pharmacological and Biomolecular Sciences, Università degli Studi di Milano, Via Balzaretti 9, 20133 Milan, Italy; 4 Dipartimento di Chimica “Ugo Schiff”, University of Florence, Via Della Lastruccia 3-13, 50019 Sesto Fiorentino, Italy

**Keywords:** nitroglycerine, CGRP, glial cells, inflammation, hyperalgesia

## Abstract

Preclinical data point to the contribution of transient receptor potential ankyrin 1 (TRPA1) channels to the complex mechanisms underlying migraine pain. TRPA1 channels are expressed in primary sensory neurons, as well as in glial cells, and they can be activated/sensitized by inflammatory mediators. The aim of this study was to investigate the relationship between TRPA1 channels and glial activation in the modulation of trigeminal hyperalgesia in preclinical models of migraine based on acute and chronic nitroglycerin challenges. Rats were treated with ADM_12 (TRPA1 antagonist) and then underwent an orofacial formalin test to assess trigeminal hyperalgesia. mRNA levels of pro- and anti-inflammatory cytokines, calcitonin gene-related peptide (CGRP) and glia cell activation were evaluated in the Medulla oblongata and in the trigeminal ganglia. In the nitroglycerin-treated rats, ADM_12 showed an antihyperalgesic effect in both acute and chronic models, and it counteracted the changes in CGRP and cytokine gene expression. In the acute nitroglycerin model, ADM_12 reduced nitroglycerin-induced increase in microglial and astroglial activation in trigeminal nucleus caudalis area. In the chronic model, we detected a nitroglycerin-induced activation of satellite glial cells in the trigeminal ganglia that was inhibited by ADM_12. These findings show that TRPA1 antagonism reverts experimentally induced hyperalgesia in acute and chronic models of migraine and prevents multiple changes in inflammatory pathways by modulating glial activation.

## 1. Introduction

Migraine is a neurovascular disorder affecting about 16% of the population worldwide [1] that displays a multifactorial etiology. Migraine disease occurs in episodes separated by headache-free days, but in 3% of patients, the number of headache days per month is 15 or more, producing a pattern of chronic migraine [2,3]. The precise pathophysiological mechanisms of the disease as well as its chronification process remain largely unknown. 

Currently, the scientific community agrees upon the activation of the trigeminovascular system as the leading cause of the generation and maintenance of migraine pain [4,5]. The activation of trigeminovascular projections induces the release of several pro-inflammatory neuropeptides and neurotransmitters in the *dura mater*, thereby inducing a cascade of inflammatory tissue responses, including vasodilation, plasma extravasation, edema, and mast cell degranulation. These events contribute to the sensitization of trigeminal neurons together with the activation of resident immune and glial cells in both peripheral (i.e., trigeminal ganglia, TGs) and central sites (i.e., trigeminal nucleus caudalis, TNC), thus activating a positive feedback loop that enhances trigeminal pain [6]. Glial cells are known to directly modulate neuronal function and activity [7,8]. The neuron–glia interactions are indeed deeply involved in all stages of inflammation and pain within the nervous system [9,10]. Clinical and preclinical evidence underscores the relationship between inflammation and migraine [6,11,12]. Inflammatory molecules may contribute to central and peripheral sensitization, thus acting as pain mediators [13,14]. In turn, central and peripheral sensitization lead to the generation of hyperalgesia and allodynia, which is associated with migraine pathogenesis [15].

The transient receptor potential ankyrin 1 (TRPA1) channels may play a role in this multifaceted scenario, as suggested by evidence provided by our group and other researchers [16,17,18,19] showing that TRPA1 antagonism is indeed effective for abolishing migraine-like changes in preclinical models. The role of TRPA1 channels in migraine pathogenesis is supported by multiple pieces of evidence. First, exogenous compounds that are TRPA1 agonists can trigger migraine attacks [20]. Furthermore, TRPA1 channels are expressed in the nerve terminals of peptidergic nociceptors [21], where their activation causes the release of calcitonin gene-related peptide (CGRP) [22]. These channels can be activated/sensitized by inflammatory components [23,24]. Finally, they are expressed not only in primary sensory neurons but also in non-neuronal cells, such as glial cells and monocytes [25,26,27,28].

TRPA1 channels may thus be a common element between pain and inflammatory pathways, and it is tempting to speculate that their manipulation may modulate both pathways with potential beneficial effects in the clinical field. In this study, we aimed to conduct an in-depth investigation of the relationship between TRPA1 channels and glial activation in migraine-associated trigeminal hyperalgesia by means of pharmacological manipulation in well-established animal models of acute and chronic migraine (see [29] for review).

## 2. Results

### 2.1. ADM_12 Counteracts Nitroglycerin (NTG)-Induced Hyperalgesia

**Figure 1:**
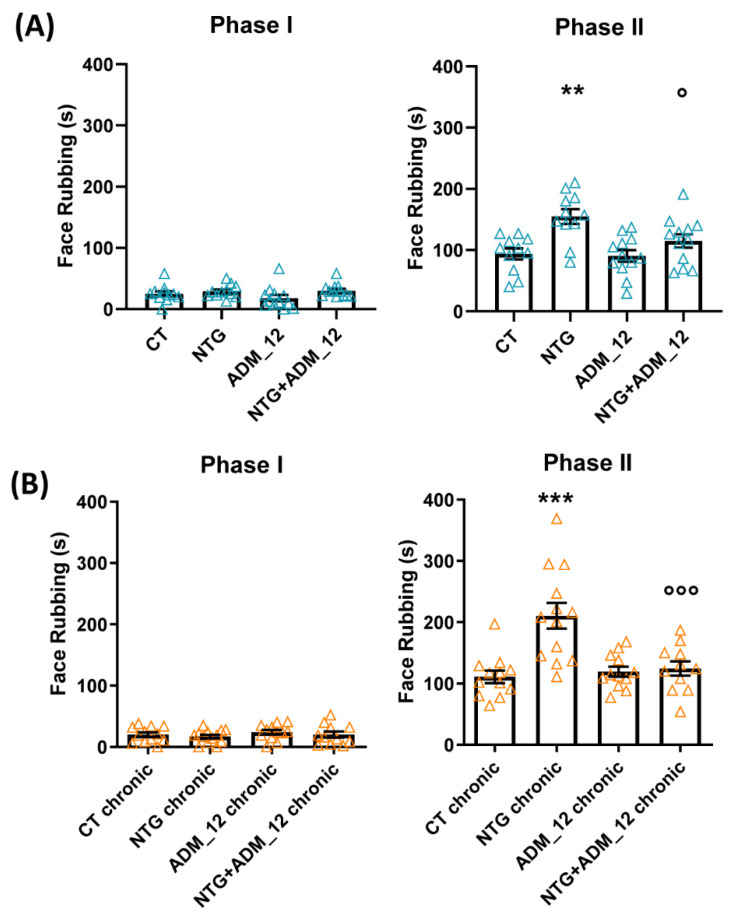
Effects of ADM_12 (30 mg/kg) in the orofacial formalin test. Face rubbing time (seconds) during Phase I and II of the orofacial formalin test in the acute migraine model (**A**) and in the chronic migraine model (**B**). CT: Control. Data are expressed as the mean ± SEM. One-way ANOVA followed by Tukey’s multiple comparisons test (*n* = 11–13 per group). ** *p* < 0.01 vs. CT and ADM_12; ° *p* < 0.05 vs. NTG; *** *p* < 0.001 vs. CT chronic and ADM_12 chronic; °°° *p* < 0.001 vs. NTG chronic.

In agreement with our previous findings [16], in the acute migraine model ADM_12 counteracted NTG-induced hyperalgesia during Phase II of the orofacial formalin test (Figure 1A). Animals treated with chronic NTG developed trigeminal hyperalgesia in Phase II of the orofacial formalin test, which was prevented by ADM_12 administration (Figure 1B). No significant differences were seen among groups during Phase I of the test in both models. These findings confirm the antihyperalgesic effect of TRPA1 blockade [16].

### 2.2. ADM_12 Effects on CGRP Gene and Protein Expression in TG

**Figure 2:**
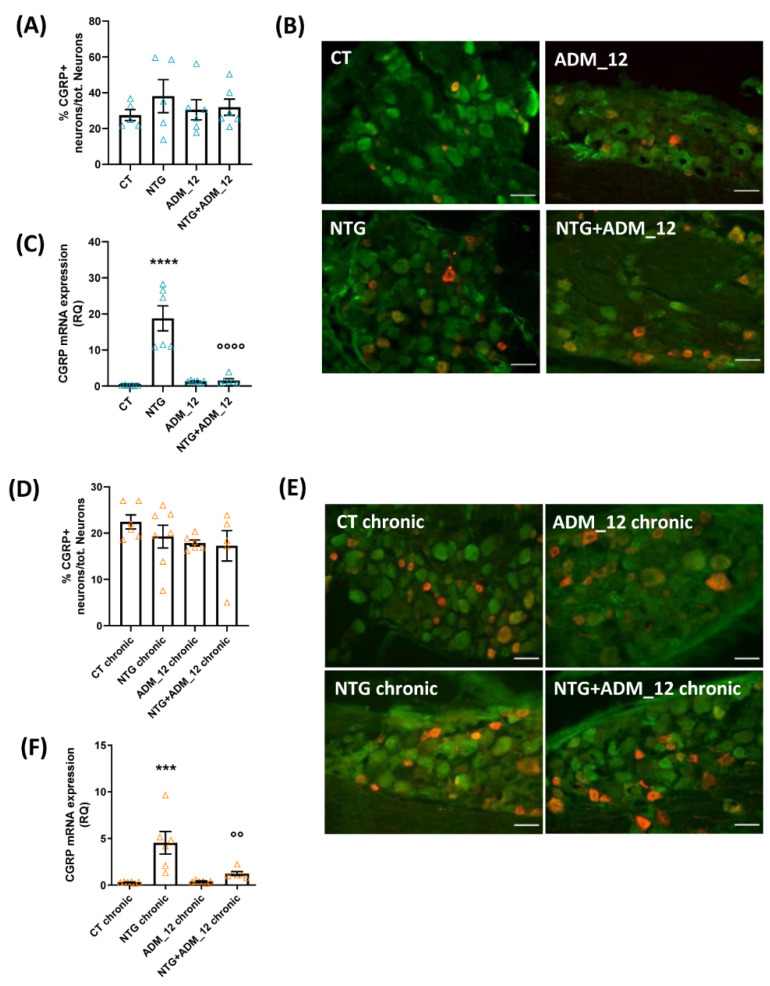
Effects of ADM_12 (30 mg/kg) on CGRP expression in the TG ipsilateral to formalin injection. Acute migraine model: (**A**) immunofluorescence analysis of the percentage of CGRP-positive neurons/tot. neurons (*n* = 5–6 per group) and representative photomicrographs (**B**). Red: CGRP; Green: NeuN; Yellow: merged. Scale bars: 50 µm. (**C**) CGRP mRNA levels, expressed as relative quantification (RQ; see Methods for details) (*n* = 6 per group). CT: Control. **** *p* < 0.0001 vs. CT and ADM_12; °°°° *p* < 0.0001 vs. NTG. Chronic migraine model: (**D**) immunofluorescence analysis of the percentage of CGRP-positive neurons/tot. neurons (*n* = 5–7 per group), and representative photomicrographs (**E**) Red: CGRP; Green: NeuN; Yellow: merged. Scale bars: 50 µm. (**F**) CGRP mRNA levels, expressed as relative quantification (RQ; see Section 4 for details) (*n* = 6 per group). *** *p* < 0.001 vs. CT chronic and ADM_12 chronic; °° *p* < 0.01 vs. NTG chronic. Data are expressed as the mean ± SEM. One-way ANOVA followed by Tukey’s multiple comparisons test.

In the TG, the percentage of CGRP-positive neurons was not significantly modified by the acute NTG challenge (Figure 2A,B). In contrast, CGRP mRNA levels were significantly increased compared with the CT group (Figure 2C). Treatment with ADM_12 markedly reduced the NTG-induced increase in CGRP mRNA levels (Figure 2C). Chronic NTG treatment did not change the percentage of CGRP-positive neurons in the TG ipsilateral to formalin injection (Figure 2D,E) but CGRP gene expression was significantly increased compared with the CT chronic group (Figure 2F). This increase was markedly reduced by ADM_12 (Figure 2F). The present data confirm the previously reported relationship between TRPA1 channels and CGRP [22].

### 2.3. ADM_12 Reduced NTG-Induced Neuroinflammation at Central and Peripheral Levels

#### 2.3.1. Effects on Inflammation-Related Glial Activation and Macrophage Cell Infiltration

**Figure 3:**
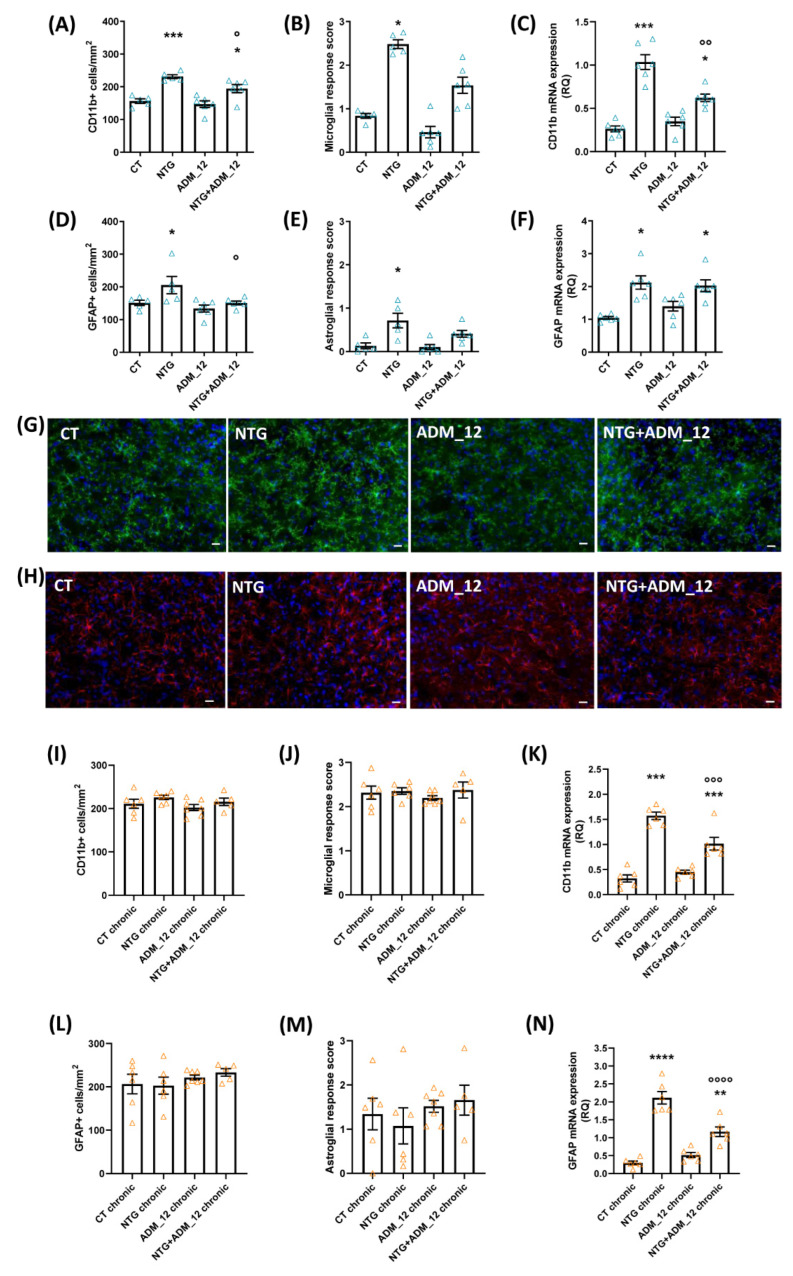
Effects of ADM_12 (30 mg/kg) on the microglial and astroglial activation ipsilateral to formalin injection. Acute migraine model (**A**–**H**): immunofluorescence analysis in the TNC area of CD11b-positive cells (**A**) and GFAP-positive cells (**D**) per area in mm^2^ (*n* = 5–6 per group). Microglial (**B**) and astroglial (**E**) response score (see Methods for details). CD11b (**C**) and GFAP (**F**) mRNA levels, expressed as relative quantification (RQ; see Section 4 for details), in the Medulla oblongata containing the TNC (*n* = 6 per group). Representative photomicrographs of microglial (**G**) and astroglial (**H**) activation; Blue: DAPI; green: CD11b; red: GFAP. Please refer to Supplementary Figure S2 for the localization of photomicrographs. Scale bars: 20 µm. CT: Control. * *p* < 0.05 and *** *p* < 0.001 vs. CT and ADM_12; ° *p* < 0.05 and °° *p* < 0.01 vs. NTG. Chronic migraine model (**I**–**N**): immunofluorescence analysis in the TNC area of CD11b-positive cells (**I**) and GFAP-positive cells (**L**) per area in mm^2^ (*n* = 5–7 per group). Microglial (**J**) and astroglial (**M**) response scores (see Methods for details). CD11b (**K**) and GFAP (**N**) mRNA levels, expressed as relative quantification (RQ; see Section 4 for details), in the Medulla oblongata containing the TNC (*n* = 6 per group). ** *p* < 0.01, *** *p* < 0.001 and **** *p* < 0.0001 vs. CT chronic and ADM_12 chronic; °°° *p* < 0.001 and °°°° *p* < 0.0001 vs. NTG chronic. Data are expressed as the mean ± SEM. One way ANOVA followed by Tukey’s multiple comparisons test for (**A**,**C**,**D**,**F**,**I**,**K**,**L**,**N**) and Kruskal–Wallis test followed by Dunn’s Multiple Comparison Test for (**B**,**E**,**J**,**M**).

In the acute migraine model, NTG administration induced microglial and astroglial activation in the TNC area that was detectable as an increased number of clusters of differentiation molecule 11b (CD11b)—(Figure 3A,G) and glial fibrillary acidic protein (GFAP)-positive cells (Figure 3D,H) when compared with the CT group. This effect was associated with a more marked activation of both glial cell populations compared with the CT group (Figure 3B,E for microglia and astrocytes, respectively). ADM_12 was able to significantly modulate NTG-induced glial activation by reducing the numbers of microglial and astroglial cells (Figure 3A,D, respectively), while the reduction pattern observed on glial cell activation did not reach a statistically significant level (Figure 3B,E for microglia and astrocytes, respectively). THe CD11b and GFAP mRNA expression levels in the TNC were significantly increased after NTG treatment (Figure 3C,F, respectively). ADM_12 significantly reduced CD11b mRNA expression (Figure 3C), while it had no effect on GFAP mRNA expression (Figure 3F).

In the chronic migraine model, the analysis of glial activation in the TNC did not show any differences among groups, either when evaluating the numbers of CD11b and GFAP positive cells (Figure 3I,L, respectively) or when considering the microglial and astroglial response scores (Figure 3J,M, respectively). In contrast, the CD11b and GFAP mRNA expression levels in the TNC area were significantly increased when compared with the CT chronic group. The CD11b and GFAP mRNA levels were significantly downregulated by ADM_12 treatment (NTG + ADM_12 group) compared with the NTG chronic group (Figure 3K,N, respectively). 

**Figure 4:**
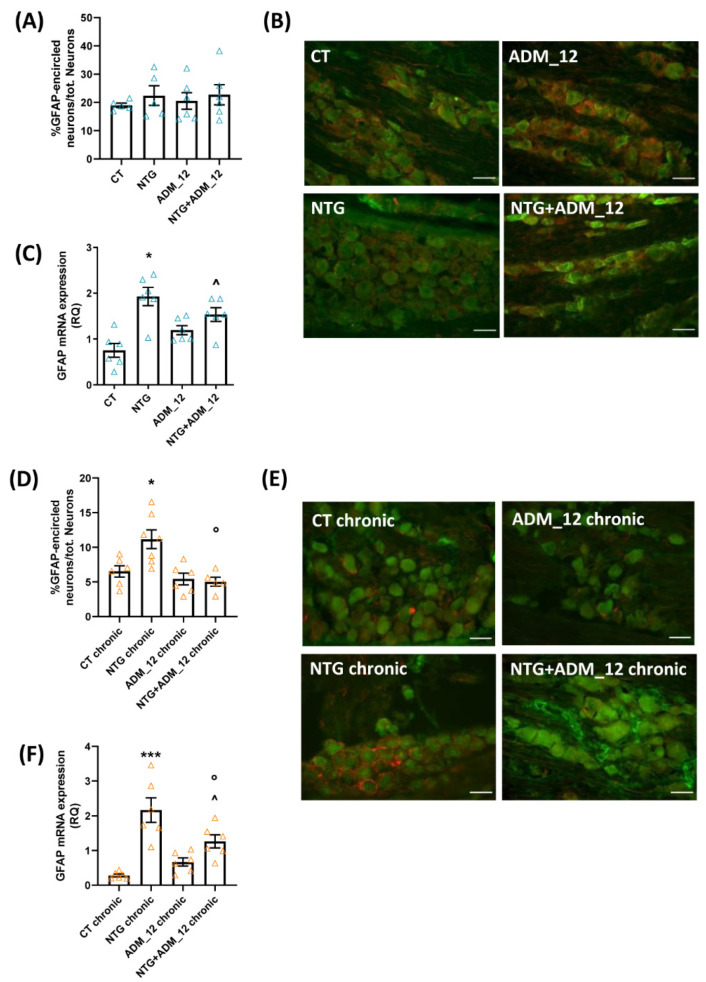
Effects of ADM_12 (30 mg/kg) on satellite glial cell activation following TG ipsilateral to formalin injection. Acute migraine model (**A**–**C**): (**A**) Satellite glial cell activation expressed as the percentage of GFAP-encircled neurons/tot. neurons (*n* = 5–6 per group) and representative photomicrographs (**B**); Red: GFAP; Green: NeuN. Scale bars: 50 µm. (**C**) GFAP mRNA levels, expressed as relative quantification (RQ; see Section 4 for details) (*n* = 6 per group). CT: Control. * *p* < 0.05 vs. CT and ADM_12; ^ *p* < 0.05 vs. CT. Chronic migraine model (**D**–**F**): (**D**) satellite glial cell activation expressed as the percentage of GFAP-encircled neurons/tot. neurons (*n* = 5–7 per group) and representative photomicrographs (**E**); Red: GFAP; Green: NeuN. Scale bars: 50 µm. (**F**) GFAP mRNA levels, expressed as relative quantification (RQ; see Section 4 for details) (*n* = 6 per group). * *p* < 0.05 and *** *p* < 0.001 vs. CT chronic and ADM_12 chronic; ^ *p* < 0.05 vs. CT chronic; ° *p* < 0.05 vs. NTG chronic. Data are expressed as the mean ± SEM. One-way ANOVA followed by Tukey’s multiple comparisons test.

Following the TG ipsilateral to formalin injection, we did not observe changes in the activation of satellite glial cells (evaluated as the percentage of neurons surrounded by GFAP-positive cells [30]) after acute NTG treatment alone or in combination with ADM_12 (Figure 4A,B) compared with the CT group. In contrast, we found a significant increase in GFAP gene expression that was not counteracted by ADM_12 treatment (Figure 4C). In contrast, chronic NTG treatment increased satellite glial cell activation as well as GFAP gene expression levels. The NTG-induced effects were counteracted by ADM_12 administration (Figure 4D–F). 

**Figure 5:**
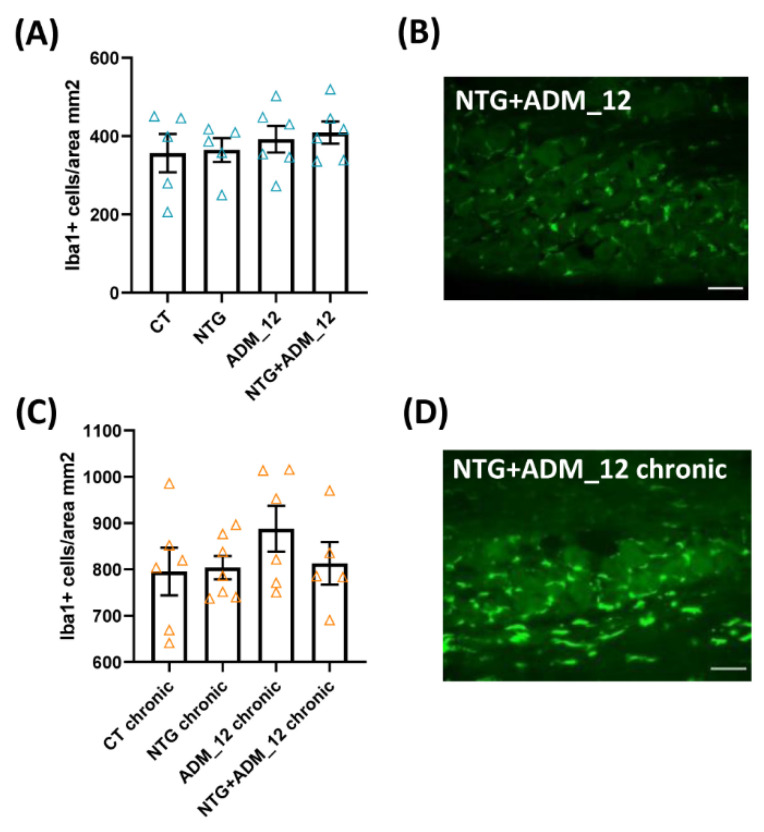
Effects of ADM_12 (30 mg/kg) on macrophage cell infiltration in the TG ipsilateral to formalin injection. Acute migraine model: (**A**) number of infiltrating macrophages (expressed as ionized calcium binding adaptor molecule 1 (Iba1)-positive cells per area in mm^2^) (*n* = 5–6 per group) and representative photomicrographs of the NTG + ADM_12 group (**B**); Green: Iba1. Scale bars: 50 µm. Chronic migraine model: (**C**) Number of infiltrating macrophages (expressed as Iba1-positive cells per area in mm^2^) (*n* = 5–7 per group) and representative photomicrograph of the NTG + ADM_12 group (**D**); Green: Iba1. Scale bars: 50 µm. CT: Control. Data are expressed as mean ± SEM. One way ANOVA followed by Tukey’s multiple comparisons test.

Regarding the number of infiltrating macrophages in the TG ipsilateral to formalin injection, no changes were reported among groups in either the acute (Figure 5A) or the chronic migraine model (Figure 5C).

In spite of the differences reported between the acute and chronic models, these data suggest the involvement of TRPA1 channels in modulating glial activation.

#### 2.3.2. Effects of ADM_12 on Pro-Inflammatory and Anti-Inflammatory Markers 

**Figure 6:**
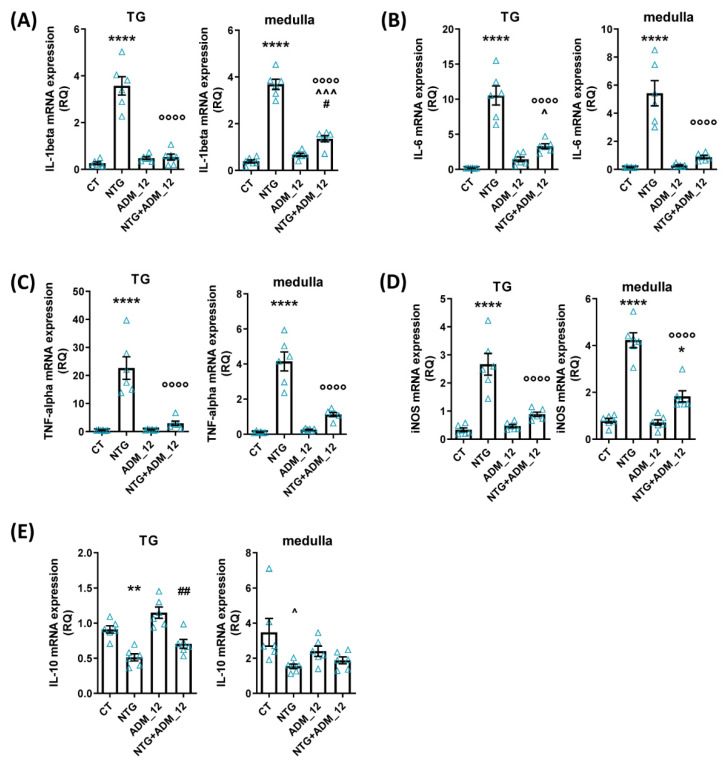
Effects of ADM_12 (30 mg/kg) on the gene expression of pro- and anti-inflammatory cytokine mediator gene expression in the Medulla oblongata and following the TG ipsilateral to formalin injection. (**A**) IL-1beta, (**B**) IL-6, (**C**) TNF-alpha, (**D**) iNOS, (**E**) IL-10 mRNA levels (expressed as relative quantification, RQ; see Section 4 for details) in the acute migraine model (*n* = 6 per group). CT: Control. * *p* < 0.05, ** *p* < 0.01 and **** *p* < 0.0001 vs. CT and ADM_12; °°°° *p* < 0.0001 vs. NTG; ^ *p* < 0.05 and ^^^ *p* < 0.01 vs. CT; # *p* < 0.05 and ## *p* < 0.01 vs. ADM_12. Data are expressed as the mean ± SEM; one way ANOVA followed by Tukey’s multiple comparisons test.

**Figure 7:**
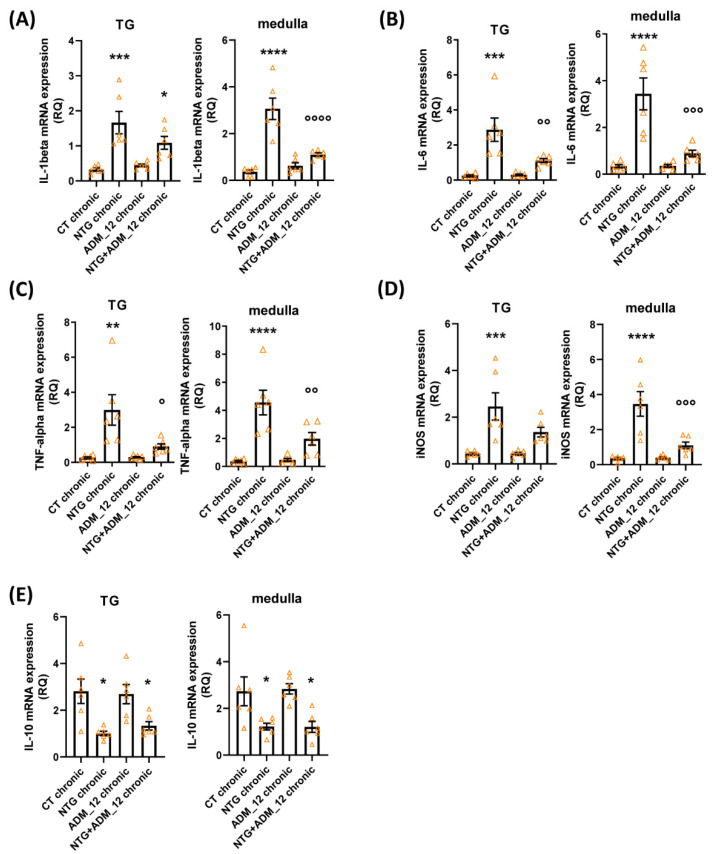
Effects of ADM_12 (30 mg/kg) on the gene expression of pro- and anti-inflammatory mediators in the Medulla oblongata and following the TG ipsilateral to formalin injection. (**A**) IL-1beta, (**B**) IL-6, (**C**) TNF-alpha, (**D**) iNOS, (**E**) IL-10 mRNA levels (expressed as relative quantification, RQ; see Section 4 for details) in the chronic migraine model (*n* = 6 per group). CT: Control. * *p* < 0.05, ** *p* < 0.01, *** *p* < 0.001 and **** *p* < 0.0001 vs. CT chronic and ADM_12 chronic; ° *p* < 0.05, °° *p* < 0.01, °°° *p* < 0.001 and °°°° *p* < 0.0001 vs. NTG chronic. Data are expressed as the mean ± SEM; one way ANOVA followed by Tukey’s multiple comparisons test.

In both the TNC area and following the TG ipsilateral to formalin injection, both acute and chronic NTG administration in the acute and chronic models caused a marked increase in the mRNA expression of the genes coding for proinflammatory markers (interleukin (IL)-1beta, IL-6, tumor necrosis factor alpha (TNF-alpha) and inducible nitric oxide synthase (iNOS)) and a significant reduction in anti-inflammatory IL-10 gene expression (Figure 6 and Figure 7, respectively). All changes in proinflammatory gene expression were significantly and markedly reduced by ADM_12 treatment in both areas under investigation (Figure 6A–D and Figure 7A–D), whereas no changes were observed in the case of the anti-inflammatory IL-10 mRNA level (Figure 6E and Figure 7E).

These results show the role of a TRPA1-related downstream signal in modulating the proinflammatory mediators in areas relevant to migraine pain.

## 3. Discussion

In the present study, we delineated the relationship between TRPA1 channels and glial activation in trigeminal hyperalgesia induced in animal models of migraine. We showed that TRPA1 antagonism is effective for reducing migraine-like pain via an interference with inflammatory pathways, thus confirming the links among TRPA1, pain, and neuroinflammation.

### 3.1. TRPA1 Antagonism in Migraine-like Pain

Acute and chronic NTG challenges lead to a trigeminal hyperalgesia that is probably caused, inter *alia*, by changes in CGRP levels. CGRP is a key mediator in the generation of migraine pain [31]. The role of CGRP at the trigeminovascular endings in the *dura mater* has been already discussed, but CGRP can also function as a positive neuromodulator in the TNC by increasing the glutamatergic activity of the second-order neurons, thus facilitating synaptic transmission and mediating central sensitization [32,33]. In the present study, we report an increase in CGRP gene expression in the TG in both the acute and chronic NTG models, although no changes in protein levels were seen by immunohistochemistry. Furthermore, we found an increase in CGRP mRNA expression but not in the CGRP protein level in the TNC area, as previously shown in the acute NTG model [16]. Regarding the chronic model, the present findings are in accordance with a previous study in which we observed an increased CGRP gene expression in different areas of the trigeminocervical complex of NTG-induced allodynic rats [34]. The discrepancy observed between gene expression and protein levels of CGRP may be related to the sample collection time and to different biological processes. We speculate that, at the time of sample collection, the neuropeptide had already been released from TG neurons (to peripheral terminals) and was no longer detectable, while the machinery associated with gene expression was captured at work by the PCR methodology. Of note, in agreement with this possible interpretation, an increased CGRP protein level in the serum of NTG-treated animals has previously been reported [35,36]. 

One of the possible mechanisms leading to CGRP release in the present models is indeed the activation of TRPA1 channels in the trigeminal nociceptive fibers [22,37]. Within the NTG model, TRPA1 activation is probably caused by either nitric oxide or reactive oxygen species and inflammatory mediators [23,38,39,40], all of which increase after the NTG challenge [17,41,42,43,44]. Accordingly, previous studies reported increased TRPA1 gene expression and immunoreactivity in the TGs of NTG-treated rats [16,45].

The most likely current scenario is that TRPA1 sensitization and CGRP release lead to the activation of the trigeminal system and the latter, in turn, enhances the transmission and perpetuation of pain signals and the release of pro-nociceptive molecules, the observed inhibitory effect of the TRPA1 antagonist in both the acute and chronic models is possibly related to the interruption of a feed-forward loop mechanism that maintains the activation of the system over time [16]. 

### 3.2. TRPA1 Antagonism and Neuroinflammation 

Besides neuropeptides, other mediators play pivotal roles in sustaining the activation of the trigeminovascular system, the hallmark of migraine pain, and neuroinflammatory molecules are indeed involved [14]. Here, both acute and chronic administration of NTG resulted in increased gene expression of pro-inflammatory cytokines and a reduction of anti-inflammatory IL-10 in the TG and TNC areas. In line with this observation, we also found increased mRNA levels of specific glial markers (both CD11b and GFAP) in the same areas. Along with the latter findings, we report an increase in glial activation (astroglia and microglia) in the TNC area, but no change in satellite glial cells in the TG within the acute model. In contrast, in the chronic model, no changes were found regarding astroglial and microglial activation in the TNC area, while increased satellite glial cell reactivity was shown in the TGs. Thus, in the chronic model, the activation of glial populations is specular to the acute NTG challenge, which suggests the occurrence of different waves of glial cell activation in the central nervous system and in the periphery as a function of time of exposure to the NTG challenge. In this context, it is possible to speculate that the expression of proteins by activated satellite glial cells may need longer procedures and/or stronger triggers to be initiated [30,46]. To the best of our knowledge, this is the first study to evaluate GFAP expression in satellite glial cells in the TG after either acute or chronic NTG administration.

In accordance with our findings, other studies using the NTG chronic model demonstrated alterations in the expression of pro-inflammatory cytokines [47,48,49,50] in the absence of changes in astrocyte densities [48]. Although, in contrast with our data, these studies reported increased microglia activation in the TNC region [48,49,50,51]. The latter discrepancy could be ascribed to differences in (i) the markers used for microglia labelling (Iba1 vs. CD11b in our study), (ii) the species used (mice vs. rats), (iii) the dose of NTG (10 vs. 5 mg/kg used in our chronic model), and (iv) the time of ex vivo assessment from the last NTG injection. Regarding the latter point, we performed our analysis 24 h after the last NTG treatment, while other studies evaluated NTG-induced effects 2 h after its administration. In this context, it must be noted that, in contrast with other types of chronic pain [52]—such as neuropathic pain—in which the temporal profile of glial activation has been carefully investigated [53,54], little is known about the timing of NTG-induced activation of glial populations. It is possible that, in a condition of repetitive, yet intermittent, NTG challenges, glial cells react by shifting their phenotype in a more rapid and dynamic way [55,56]. Thus, at 24 h from the last NTG administration, changes in glial activation are no longer visible, while increased expression of pro-inflammatory cytokines is still evident. In addition, in the TG, increased reaction of satellite glial cells is also appreciable during the chronic challenge, suggesting a process that is continuously responding to NTG, as are the pro-inflammatory agents.

We did not detect changes in the amount of macrophage infiltration in the TG in either model. While we cannot rule out the occurrence of macrophage activation at intermediate time-points between the single and the chronic administration protocols adopted, the most likely explanation is that macrophages in the TG do not significantly contribute to NTG-triggered trigeminal sensitization in contrast with what has been observed in response to stronger inflammatory stimuli [30,46,57] or in neuropathic pain models or, again, in a genetic mouse model of migraine [58].

Despite the abovedescribed differences in the acute and the chronic models, it is evident that NTG does activate glial cells. An in vitro study reported that the application of NTG induced an increase in NO production in astroglial and microglial cells as well as an increase in iron trafficking [59]. The high intracellular concentration of iron in glial cells can trigger oxidative stress—known to be involved in migraine pathophysiology [60]—which, in turn, can activate and stimulate TRPA1 channels [39]. It is therefore possible that this mechanism, in concert with other pathways recruited following NTG administration [29], may contribute to the activation of glial cells through TRPA1 channels expressed by astrocytes [61,62] and satellite glial cells [25]. Even though confirmatory data are still needed, the function of TRPA1 channels in these cells lies in their ability to act as regulators of basal calcium levels [25,27]. It has been suggested that astrocytic calcium hyperactivity may influence glutamatergic synaptic function [63]. Indeed, calcium dynamics appear to be linked with adjacent neuron interactions [64]. It is interesting to note that TRPA1 channels appear to be only marginally involved in the calcium signal of astrocytes under physiological conditions, while their role becomes more prominent under pathological conditions [63]. This hypothesis is in line with the characteristics of these channels when responding to different types of harmful stimuli [65], e.g., the NTG-induced inflammation in the present study. We previously showed that NTG increases TRPA1 gene expression in the TG and TNC area [16]. The enhancement of TRPA1 expression leads to further cytokine release (e.g., IL-6) [24], which is in accordance with the present data. This effect may be promoted by hypoxia-inducible factor-1alpha through nuclear factor-kappa B (NF-kB) [24], whose activation is enhanced by NTG [43,44,66]. NF-kB may also induce the activation of microglia and astrocytes [67] with the consequent release of pro-inflammatory agents, leading to inflammatory pain and contributing to hyperalgesia [68,69]. 

Microglia as well was activated by the NTG challenge. Since the microglia do not express TRPA1 channels, their activation is probably due either to the microglia–astroglia interaction [70] or to crosstalk between the microglia/astroglia and neurons [71,72]. Among the numerous agents that mediate microglia/astroglia/neuron crosstalk, a significant role is played by cytokines [70]. In particular, in a model of neuropathic pain, microglia–neuron crosstalk was shown to be mediated by IL-6 [71], whose expression was found to be increased in NTG-challenged animals, together with other pro-inflammatory molecules, namely IL-1beta, TNF-alpha, and iNOS. 

Besides the inflammatory-related agents, nitric oxide, and reactive oxygen species, it is also possible that TRPA1 channels located in TG neurons are activated by toll-like receptor 7 (TLR7), which are expressed in TG as well [73], through a functional interaction [74]. TLR7 receptors, like other members of the TLR family, act as sensors of exogenous and endogenous damage signals and are deeply involved in the generation and maintenance of inflammatory pain. Their engagement may stimulate intracellular signaling pathways, leading to the synthesis and secretion of inflammatory cytokines [75].

**Figure 8:**
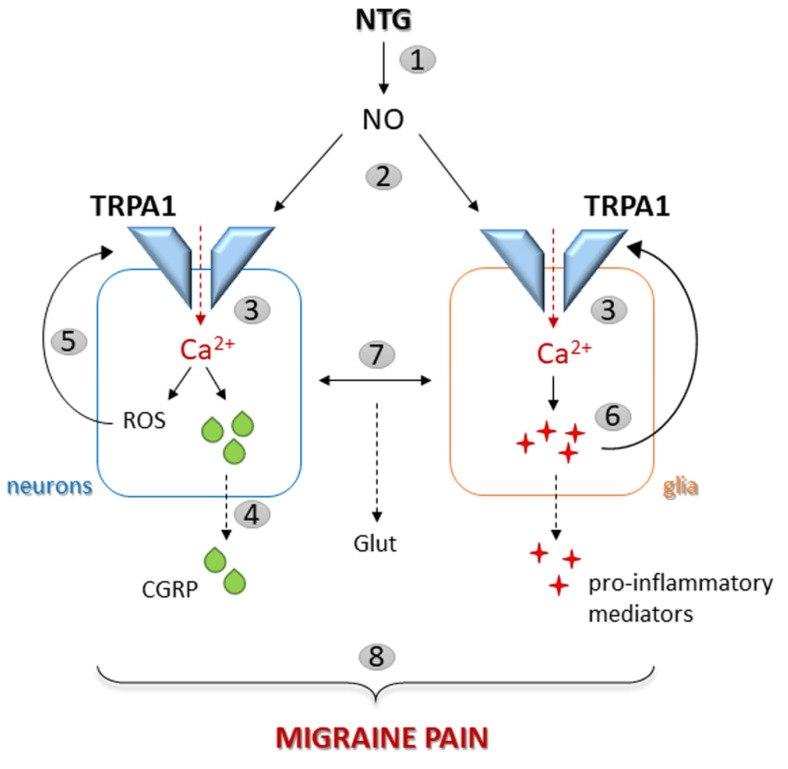
Schematic representation of the putative TRPA1-mediated mechanisms involved in migraine pain. The administration of NTG promotes the formation of nitric oxide (NO) (1) which directly activates TRPA1 channels (2). Once activated, these channels allow the intracellular concentration of calcium to increase (3). At the neuronal level, TRPA1 activation promotes the release of CGRP (4) and the generation of reactive oxygen species (ROS), which can sensitize the TRPA1 channels (5). In glial cells, following TRPA1 activation and calcium entrance (3), the expression of glial markers and pro-inflammatory molecules (sensitizing TRPA1 channels) increases (6) and neuron–glia coupling promotes glutamatergic signaling (Glut) (7). All of these processes contribute to the generation and maintenance of migraine pain (8).

Of note, all the above-mentioned responses induced by NTG were reduced/abolished by a single treatment with ADM_12, meaning that TRPA1 channels are involved in mediating glial inflammatory pathways linked to migraine pain (Figure 8). It is therefore possible to speculate that nitric oxide released/produced from NTG may activate several pathways that lead to trigeminovascular system activation [17], including the activation of TRPA1 channels [76]. Once activated, these channels induce an increase in the intracellular concentration of calcium. At the neuronal level, i.e., on the trigeminal nociceptors, TRPA1 activation promotes the release of the neuropeptide CGRP, thus starting the cascade of molecular mechanisms that lead to neurogenic inflammation and migraine pain. In addition, the intracellular calcium rise evokes the generation of reactive oxygen species in the soma of TG neurons [17], which can further sensitize the TRPA1 channels, thus perpetuating the nociceptive processing. As extensively reported above, following the activation of TRPA1 located on glial cells and calcium entrance, the expression of glial and pro-inflammatory markers increases (in turn activating/sensitizing TRPA1), and the neuron–glia coupling promotes glutamatergic signaling [27].

In this scenario, the key role of TRPA1 channels, whose blockade may abolish calcium hyperactivity [63] and all the downstream mechanisms, thus interrupting the positive feedback loop that causes the release of neuroactive peptides (e.g., CGRP) [42] and pro-inflammatory mediators involved in migraine-related pain [77], is evident.

### 3.3. Limitations of the Study and Future Directions

This study produced intriguing data but, at the same time some open-ended questions remain. First, there is a need to understand the activation pattern of the mechanisms and pathways underlying the acute and chronic NTG models. A time-course analysis would probably better inform us about the differences and similarities between the two NTG models that emerged from this study. Second, it would also be interesting to confirm the present data without the possible confounding effects of formalin injection. It is known, indeed, that formalin can activate the TRPA1 channels [78]; however, we did not find any effects in the orofacial formalin test with the sole use of ADM_12 on basal nociception. Here, we used 1.5% (*v*/*v*) formalin, which is the lowest percentage able to induce hyperalgesia [79], while, in other studies that showed the ability of the TRPA1 antagonist to counteract formalin-induced pain, the percentage used was higher (2.5 or 5% *v*/*v*) [78,80]. In addition, Fischer et al. [81] reported, in vitro, that formalin can also activate a TRPA1-independent pathway. Another reason for such results may be possibly due to the properties of the antagonists used. 

Despite these observations, it should be noted that the TRPA1 channels have been implicated in NTG-induced pain, as confirmed by other studies [17,18,19]. Nonetheless, it will be necessary to define the precise mechanisms through which TRPA1 channels mediate the neuroinflammatory response in the context of migraine-like pain. Third, there is a need to delineate a time course of the occurring events, together with an analysis of TRPA1 channel expression and functions. Indeed, even though the ability of ADM_12 to inhibit the TRPA1 channels activity has previously been reported [82], it would be of great relevance to directly investigate the activity and functionality of TRPA1 channels in relation to the present migraine models. Additionally, notwithstanding the great amount of available literature supporting our findings about TRPA1 involvement in migraine, our data may benefit from validation with different TRPA1 antagonists. In this context, it is of paramount importance to include female animals in future studies due to the fact that women are more prone to the development of migraine pain than men, as it is also influenced by the estrous cycle. It should be noted, however, that some studies have shown no differences between male and female animals in the NTG model (even in chronic conditions) [83,84], probably due to the test used.

It is interesting to note that a single administration of ADM_12 was able to abolish the NTG-induced effects, even in the chronic model. A similar result was previously reported by our group in a different model of chronic pain, where a single treatment with ADM_12 reduced trigeminal allodynia in rats with trigeminal neuropathic pain [85]. These encouraging findings leave questions about the possibility that chronic TRPA1 antagonism would have completely prevented the development of NTG-induced hyperalgesia and the different responses obtained in the chronic model unanswered. In a different area of research, it was demonstrated that TRPA1 antagonism can prevent the transition from acute to chronic inflammation and pain in a model of chronic pancreatitis by reducing neurogenic inflammation [86]. Additionally, in a model of peripheral diabetic neuropathy, prolonged blockade of TRPA1 delayed the loss of nociceptive nerve endings, thus suggesting this strategy as a possible disease-modifying treatment [87,88].

## 4. Materials and Methods

### 4.1. Animals

For this study, we used a total of 94 adult male Sprague-Dawley rats (weight 200–250 g, Charles River Laboratories, Como, Italy). Animals were housed in plastic boxes in groups of two with water and food available ad libitum and kept on a 12:12 h light–dark cycle. All procedures were approved by the Italian Ministry of Health (Document number 1239/2015-PR and 1032/2015-PR) and performed in agreement with the guidelines of the European Community Directive 2010/63/EU of 22 September 2010. Upon arrival, animals were habituated to the housing conditions for 1 week before the experimental testing. 

### 4.2. Experimental Plan

The acute and chronic migraine-like models were based on the administration of NTG (Bioindustria L.I.M. Novi Ligure (AL), Italy) dissolved in 27% alcohol and 73% propylene glycol as a stock solution of 5.0 mg/1.5 mL. For the injections, NTG was further diluted in saline (0.9% NaCl) to reach the final concentration of 6% alcohol and 16% propylene glycol. In the acute migraine model, NTG was administered as a single injection intraperitoneally (i.p.) at a dose of 10 mg/kg 4 h before testing [89]. For the chronic model, NTG was injected i.p. at a dose of 5 mg/kg every other day over a 9-day period for a total of five injections [34] before testing (Supplementary Figure S1).

The TRPA1 antagonist ADM_12 [82] was dissolved in saline and administered i.p. at a dose of 30 mg/kg [16] administered 3 h after NTG/vehicle treatment in the acute model [16] or 24 h after the last NTG injection in the chronic model. One hour after ADM_12/saline treatment, within either the acute or the chronic model, animals underwent the orofacial formalin test. At the end of the test, rats were euthanized, and the nervous tissue samples were collected and processed for immunofluorescent analyses or for Real Time PCR quantification.

The experimental timeline for the treatment and testing procedures is shown in Supplementary Figure S1, while the experimental groups and sets are illustrated in Table 1. Of note, each group received two types of treatment: NTG or its vehicle (6% alcohol + 16% propylene glycol and saline) with an acute or chronic frequency depending on the model and ADM_12 or its vehicle (0.9% saline). Thus, rats in the CT group received both the NTG vehicle and saline (Table 1).

### 4.3. Orofacial Formalin Test 

In association with the NTG models, which are able to induce cephalic and extracephalic changes [29], in the present study we used the orofacial formalin test to specifically activate the trigeminal system. 

The behavioral test was performed as described in detail elsewhere [89,90,91]. Briefly, after acclimatization (20 min) to the test chamber, rats were injected subcutaneously with 50 μL of 1.5% formalin (made of formaldehyde 37% in water and 0.9% saline, *v*/*v*) into the right upper lip. Face rubbing was measured by a researcher blind to group assignment who counted the seconds spent by the animal grooming the injected area with the ipsilateral forepaw or hind paw during the following periods: minutes 0–3 (Phase I) and minutes 12–45 (Phase II) after formalin injection.

### 4.4. Immunofluorescence Staining

Forty-five minutes after formalin injection, the animals were anaesthetized (Sodium thiopental, 150 mg/kg, i.p.) and transcardially perfused with saline and 4% paraformaldehyde. The medullary segment containing the TNC between +1 and −5 mm from the obex was removed, post-fixed for 24 h in the same fixative and subsequently transferred to solutions of sucrose at increasing concentrations (up to 30%) during the following 72 h. All samples were cut transversely to a thickness of 30 µm on a freezing sliding microtome. The TGs were removed, post-fixed for 1 h in the same fixative, and then rinsed in PBS and transferred in a 30% sucrose solution for 24 h. TG samples were then embedded in mounting medium (OCT; Tissue Tek, Sakura Finetek, Zoeterwoude, The Netherlands) and kept at −80 ℃ until use. TG sections were cut longitudinally to a thickness of 20 µm using a cryostat. 

For the assessment of microglial and astroglial immunofluorescent labeling, the TNC sections were processed according to the following steps: (1) mounted on slides, dried at room temperature for 30 min, and washed with PBS; (2) blocked for 1 h at room temperature in PBS containing 10% normal goat serum (NGS) and 0.3% Triton X-100; (3) incubated overnight at 4 °C in PBS/1% NGS/0.3% Triton X-100 with mouse anti-CD11b (1:300, Serotec MCA275R) primary antibody (to detect microglia) or rabbit anti-GFAP (1:500, Dako, Glostrup, Denmark) primary antibody (to label astrocytes); (4) rinsed in PBS and incubated for 1 h at room temperature in PBS/1% NGS with goat anti-mouse AlexaFluor^®^ 488 or goat anti-rabbit AlexaFluor^®^ 594 secondary antibodies (1:300, Life Sciences, Monza, Italy); and (5) rinsed in PBS and covered with Fluoroshield^TM^ DAPI (Sigma-Aldrich, Merk, Milano, Italy) to label cell nuclei.

The evaluation of activated satellite glial cells, macrophage infiltration [30], and CGRP expression in TGs was performed as follows: (1) sections were blocked for 45 min at room temperature in PBS containing 10% NGS and 0.1% Triton X-100; (2) incubated overnight at 4 °C with the following rabbit primary antibodies: rabbit antibodies against the microglia marker Iba1 (anti-Iba1, 1:500; Wako, Osaka, Japan), the astrocyte/satellite glial cell marker GFAP (anti-GFAP, 1:600; Dako, Glostrup, Denmark), and CGRP (anti-CGRP, 1:500; Enzo Life Sciences, Farmingdale, NY, USA), and mouse primary antibodies antibody against the neuronal nuclei marker NeuN (anti-NeuN, 1:150; Chemicon, Darmstadt, Germany); (3) Sections were rinsed three times with PBS and incubated for 1 h at room temperature with the AlexaFluor^®^ 488- or AlexaFluor^®^ 555-conjugated secondary antibodies (1:600; Life Technologies, Carlsbad, CA, USA). All antibodies were diluted in PBS containing 0.1% Triton X-100 and 5% NGS; (4) Cell nuclei were counterstained with the Hoechst33258 dye (1:20,000; Sigma-Aldrich, Merck, Milano, Italy); (5) Sections were rinsed in PBS and mounted with Dako Fluorescent mounting medium.

### 4.5. Image Analysis

In the TNC area, image analysis was performed using an AxioSkop 2 microscope (Zeiss) and a computerized image analysis system (AxioCam, Zeiss, Milan, Italy) equipped with dedicated software (AxioVision Rel 4.2, Zeiss, Milan, Italy). The cell counts for microglia and astroglia were assessed by counting CD11b- or GFAP-positive cells, respectively, from a stack of 16 pictures (1 μm-thick, 20× magnification) taken from four representative sections alongside the TNC following the rat brain atlas coordinates (bregma, −14.08 to −14.60 mm; Paxinos and Watson 4th edition). The total numbers of CD11b- and GFAP-positive cells of the four sections were summed and expressed per mm^2^. The location of IF images in the TNC area is reported in Supplementary Figure S2.

The glial activation state within the TNC was detected by a qualitative analysis conducted using a modified version of the Colburn microglia and astroglia response scale [92]. The glial activation state was rated by a researcher blind to group assignment on a scale ranging from 0 to 3 [93], where 0 = resting state with long thin projections and well-spaced cells; 1 = mild response in which projections are still branched and there is less space between cells; 2 = moderate response with less ramified projections, an increased cell density, and cells occasionally overlapping; and 3 = intense response with few and short projections and densely arranged/extensively overlapping cells. In case of no clearly defined response, intermediate values were used (i.e., 0.5, 1.5, 2.5). The final activation state was calculated as the average of the scores assessed for each of the four representative sections of the TNC. 

In the TGs, the number of Iba1+ cells/area and the percentages of GFAP-encircled neurons and CGRP+ neurons were assessed in whole TG sections acquired at 20× magnification using an inverted fluorescence microscope (200M; Zeiss, Milan, Italy) connected to a PC computer equipped with AxioVision software (Zeiss, Milan, Italy).

Image analysis was performed by a researcher blind to group assignment.

For both the acute and chronic NTG models, no differences in the immunofluorescence analysis were reported between the sides ipsi- and contralateral to formalin injection of TNC and TGs (Supplementary Figures S3–S6). Thus, only immunohistochemical data and analysis of the ipsilateral side were considered for the analysis and reported.

### 4.6. Real-Time PCR

At the end of the behavioral test, rats were euthanized with a lethal dose of anesthetic (Sodium thiopental, 150 mg/kg, i.p.). Then, the Medulla oblongata (bregma, −13.30 to −14.60 mm) and TG ipsilateral to the formalin injection were quickly dissected out, rinsed in cold sterile saline solution, placed in cryogenic tubes, and immediately frozen in liquid nitrogen. The gene expression analysis was performed only on the ipsilateral side to the formalin injection, since no differences between ipsi- and contralateral sides were reported in the immunofluorescence analysis (see above).

The collected areas were kept at −80 °C until processing. The mRNA expression levels of the genes coding for TNF-alpha, IL-1beta, IL-6, IL-10, iNOS, GFAP, and CGRP (whose primer sequences are reported in Supplementary Table S1) were measured by Real Time-PCR, as previously reported [16,89,91]. Glyceraldehyde 3-phosphate dehydrogenase (GAPDH), whose expression remained constant in all experimental groups, was used for normalization. All samples were assayed in triplicate; gene expression levels were calculated and expressed as the relative quantification (RQ) according to the 2^−∆∆Ct^ = 2^−(∆Ct gene − ∆Ct housekeeping gene)^ formula by using Ct (cycle threshold) values.

### 4.7. Statistical Evaluation

Based on previous studies [16,94] a power analysis was conducted (GPower 3.1) to calculate the minimal sample size needed to obtain a statistical power of 0.8 at an alpha level of 0.05. We hypothesized a difference of at least 20% in the total nociceptive response during the second phase of the orofacial formalin test (face rubbing time) between rats injected with NTG and rats injected with the NTG vehicle, thus estimating a sample size of 6 rats in each experimental group with an effect size of 1.91 with a maximum of 7 rats per group considering the variability of test.

The statistical analysis was performed with the GraphPad Prism program (version 8, GraphPad Software, San Diego, CA, USA). All data were tested for normality using the Kolmogorov–Smirnov normality test and considered normal. Differences between groups were analyzed by one-way analysis of variance (ANOVA) followed by Tukey’s multiple Comparison Test or the Kruskal–Wallis test followed by Dunn’s Multiple Comparison Test (used for glial response score). Differences between ipsilateral and contralateral sides were analyzed with two-way ANOVA followed by Sidak’s multiple comparisons test. A probability level of less than 5% was regarded as significant.

## 5. Conclusions

Great steps are being taken in the advancement of migraine pathophysiology characterization, although many of the mechanisms underlying this complex neurological disease remain to be elucidated. The findings of the present study contribute important information by showing that glial activation via TRPA1 channels may contribute to trigeminal hyperalgesia associated with migraine-like pain.

## Figures and Tables

**Table 1:** Experimental groups within the acute and chronic NTG models with details of treatments and the number (N) of animal per group assigned to different experimental procedures and analyses.

	Group Name	NTG/Control Treatment (i.p. Injection)	ADM_12/Control Treatment (i.p. Injection)	OFT (N)	Real Time- PCR (N)	IF (N)
ACUTE MODEL	CT	acute NTG vehicle	saline	11	6	5
	NTG	acute NTG (10 mg/kg)	saline	11	6	5
	ADM_12	acute NTG vehicle	ADM_12 (30 mg/kg)	12	6	6
	NTG + ADM_12	acute NTG (10 mg/kg)	ADM_12 (30 mg/kg)	12	6	6
CHRONIC MODEL	CT chronic	chronic NTG vehicle	saline	12	6	6
	NTG chronic	chronic NTG (5 mg/kg)	saline	13	6	7
	ADM_12 chronic	chronic NTG vehicle	ADM_12 (30 mg/kg)	12	6	6
	NTG + ADM_12 chronic	chronic NTG (5 mg/kg)	ADM_12 (30 mg/kg)	11	6	5

IF = immunofluorescence analysis; OFT = orofacial formalin test.

## Data Availability

The datasets reported in the current study are openly available in the ZENODO repository, (https://doi.org/10.5281/zenodo.7030839).

## Supplementary Materials

The following supporting information can be downloaded at: https://www.mdpi.com/article/10.3390/ijms232214085/s1.
