# Supplementary material for: Modulation of Glia Activation by TRPA1 Antagonism in Preclinical Models of Migraine
Source: Int J Mol Sci. 2022 Nov 15;23(22):14085. doi: 10.3390/ijms232214085 (PMC9697613; doi:10.3390/ijms232214085)
Supplement: Supplementary file 1 [file ijms-23-14085-s001.zip › ijms-1997431-supplementary.pdf]

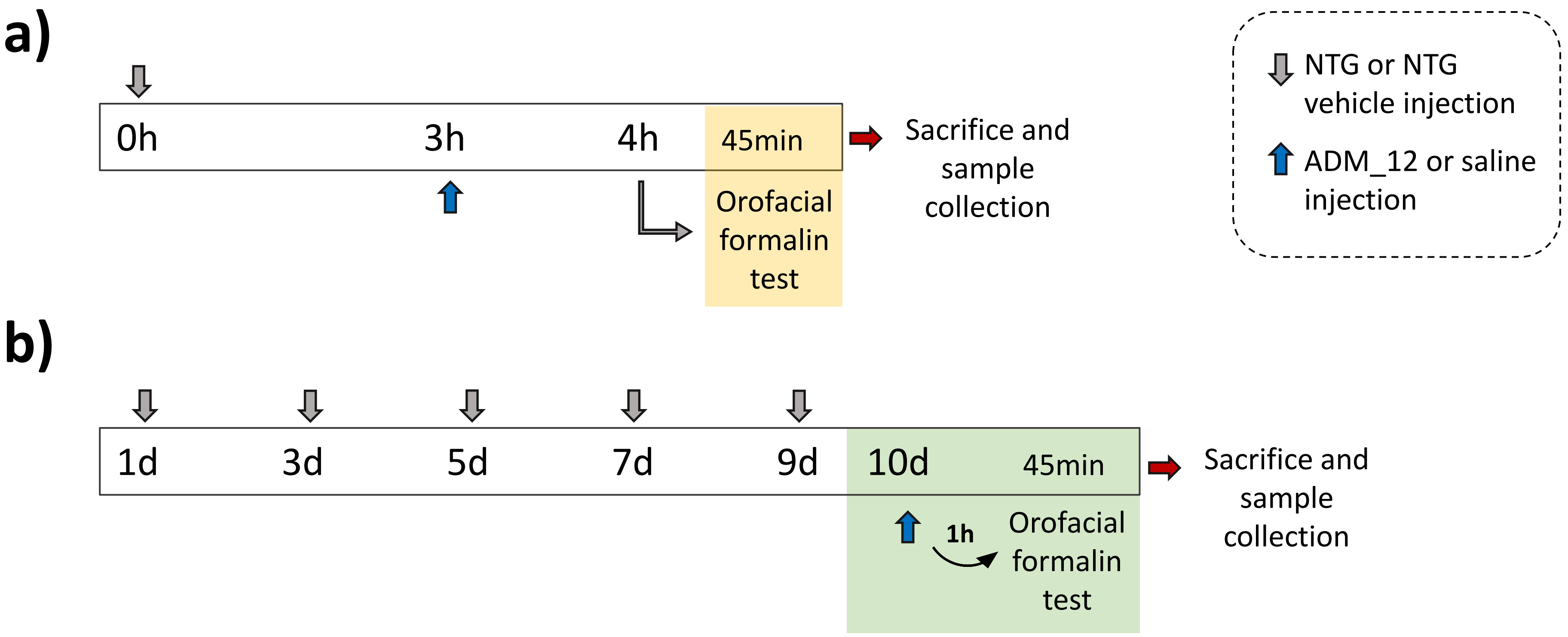

Figure S1. Experimental timeline for treatment and testing procedures. (**a**) acute migraine model: ADM_12 (or its vehicle) was administered 3h after NTG (or its vehicle) and 1h later (i.e., 4h from NTG/vehicle injection) rats underwent the orofacial formalin test (45 min duration). At the end of the test, the TNC area and the TGs were collected for ex vivo analysis. (**b**) chronic migraine model: NTG (or its vehicle) was administered every other day over a 9-day period; 24 h after the last NTG/vehicle injection, rats were treated with ADM_12/saline and 1 h later underwent the orofacial formalin test (45 min duration). At the end of the test, the TNC area and the TGs were collected for ex vivo analysis.

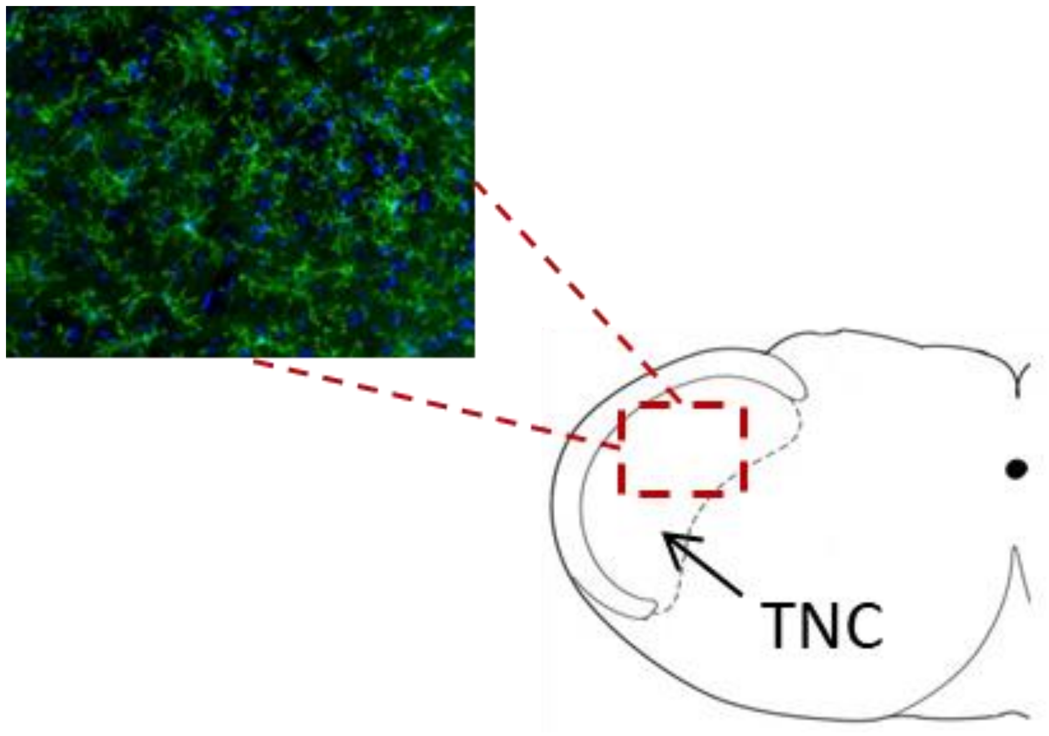

Figure S2. Location of the IF images in the TNC area. Site of acquisition of a representative photomicrograph at 20x magnification within the TNC. Green: CD11b staining of microglia; Blue: cell nuclei stained with DAPI.

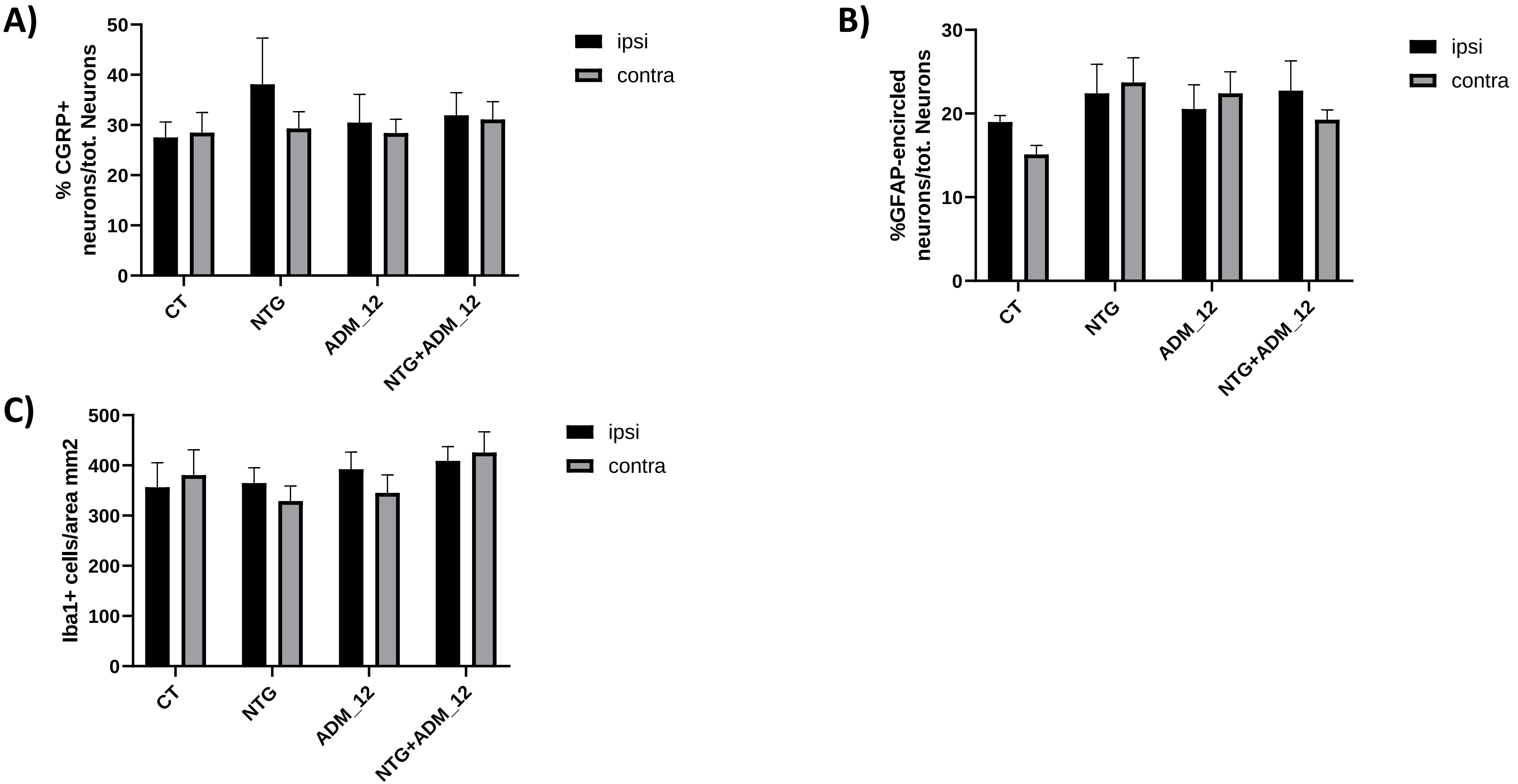

Figure S3. Acute NTG model: comparison of immunofluorescence analysis data in the ipsi- and contralateral (with respect to formalin injection) TGs. (**A**) Percentage of CGRP-positive neurons/tot. neurons. (**B**) Satellite glial cell activation expressed as %of GFAP-encircled neurons/tot. neurons (**C**). Number of infiltrating macrophages expressed as Iba1-positive cells per area in mm^2. Data are expressed as mean ± SEM. No statistically significant differences have been detected. Two-way ANOVA followed by Sidak's multiple comparisons test.

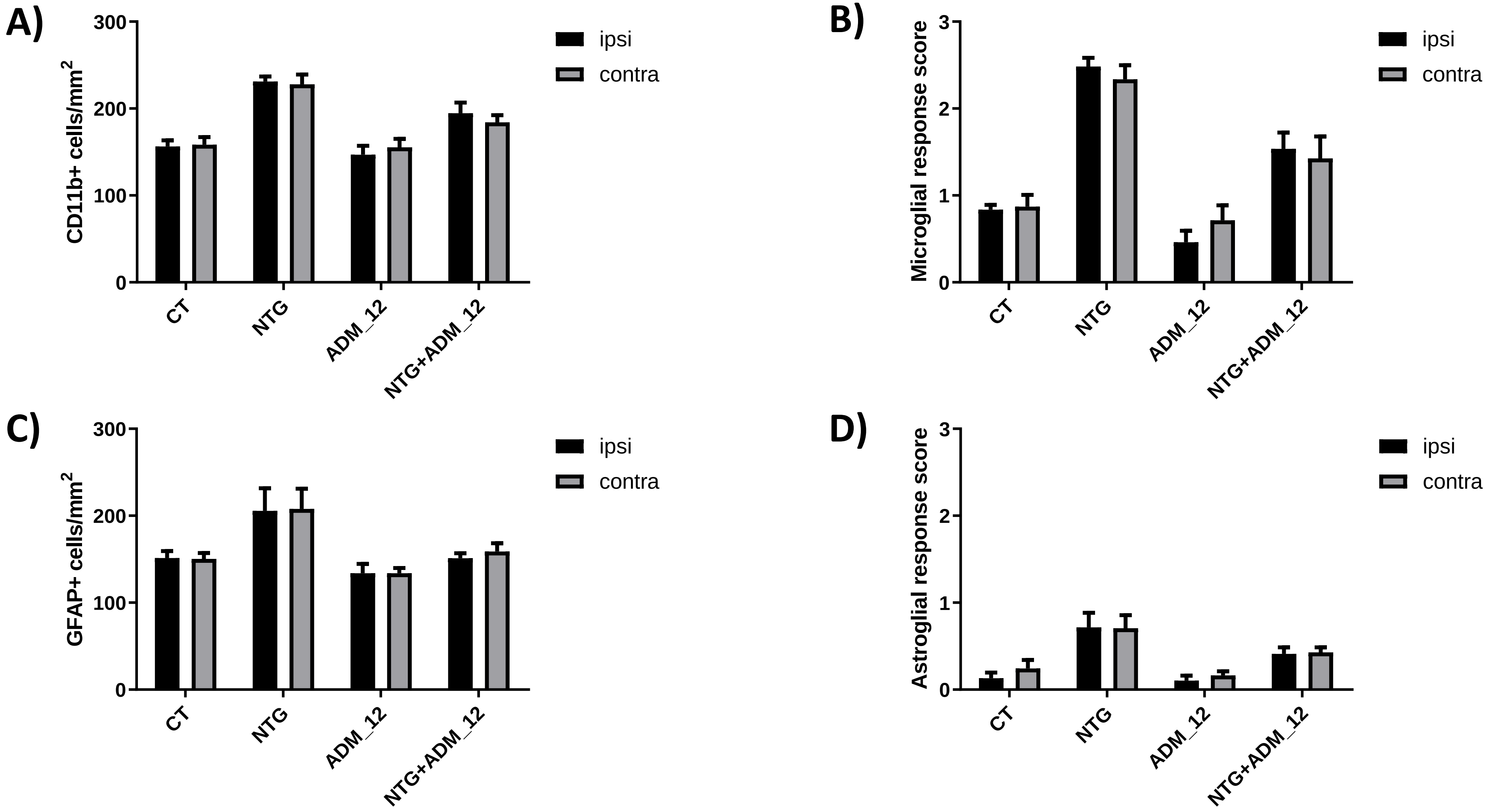

Figure S4. Acute NTG model: comparison of immunofluorescence analysis data ipsi- and contralateral (with respect to formalin injection) TNC. Immunofluorescence analysis of the CD11b-positive cells per area in mm^2 (**A**) and microglial response score (**B**). Immunofluorescence analysis of the GFAP-positive cells per area in mm2 (**C**) and astroglial response score (**D**). Data are expressed as mean ± SEM. No statistically significant differences have been detected. Two-way ANOVA followed by Sidak's multiple comparisons test.

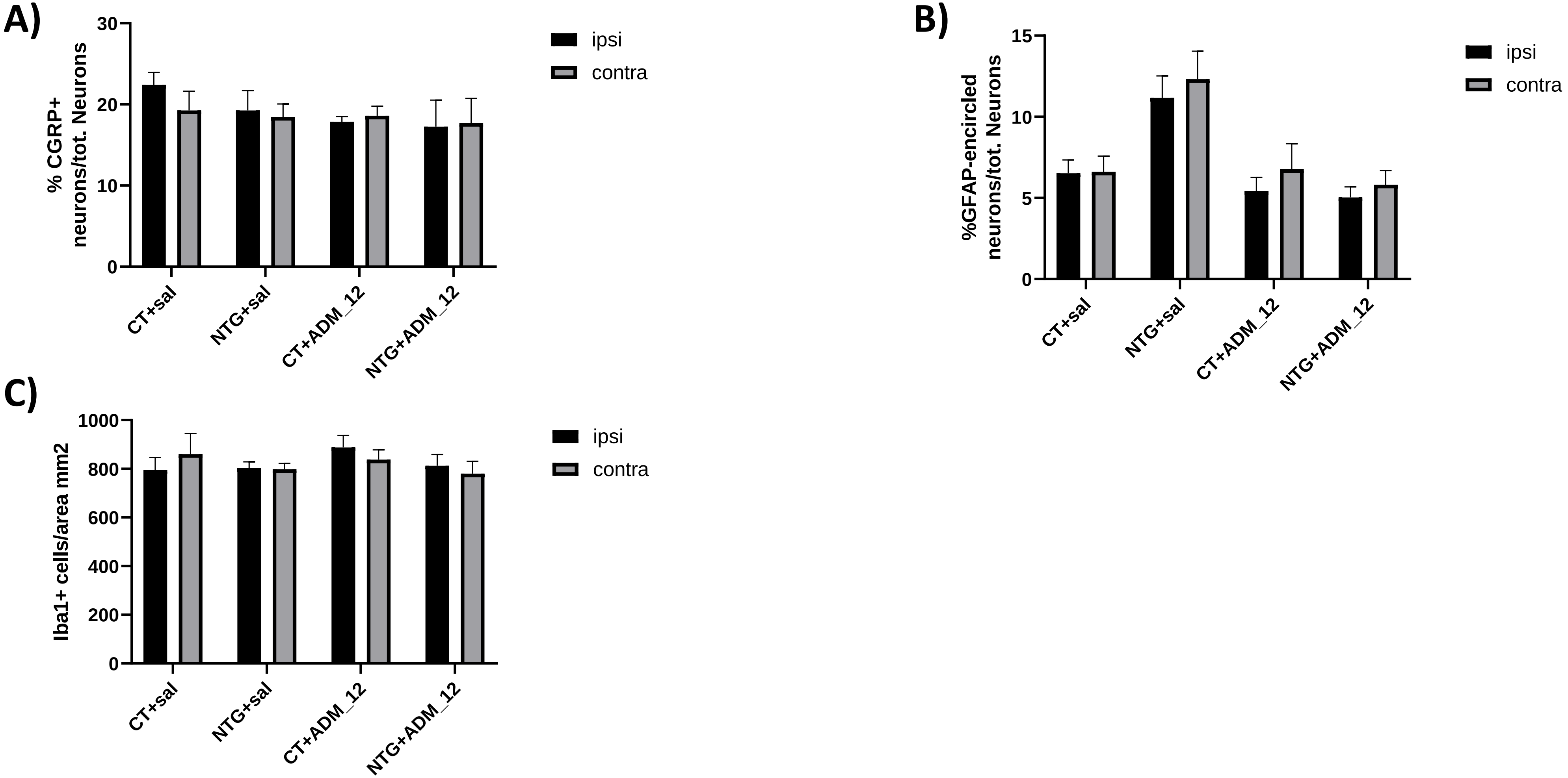

Figure S5. Chronic NTG model: comparison of immunofluorescence analysis data ipsi- and contralateral (with respect to formalin injection) TGs. (**A**) Percentage of CGRP-positive neurons/tot. neurons. (**B**) Satellite glial cell activation expressed as % of GFAP-encircled neurons/tot. neurons (**C**). Number of infiltrating macrophages expressed as Iba1-positive cells per area in mm^2. Data are expressed as mean ± SEM. No statistically significant differences have been detected. Two-way ANOVA followed by Sidak's multiple comparisons test.

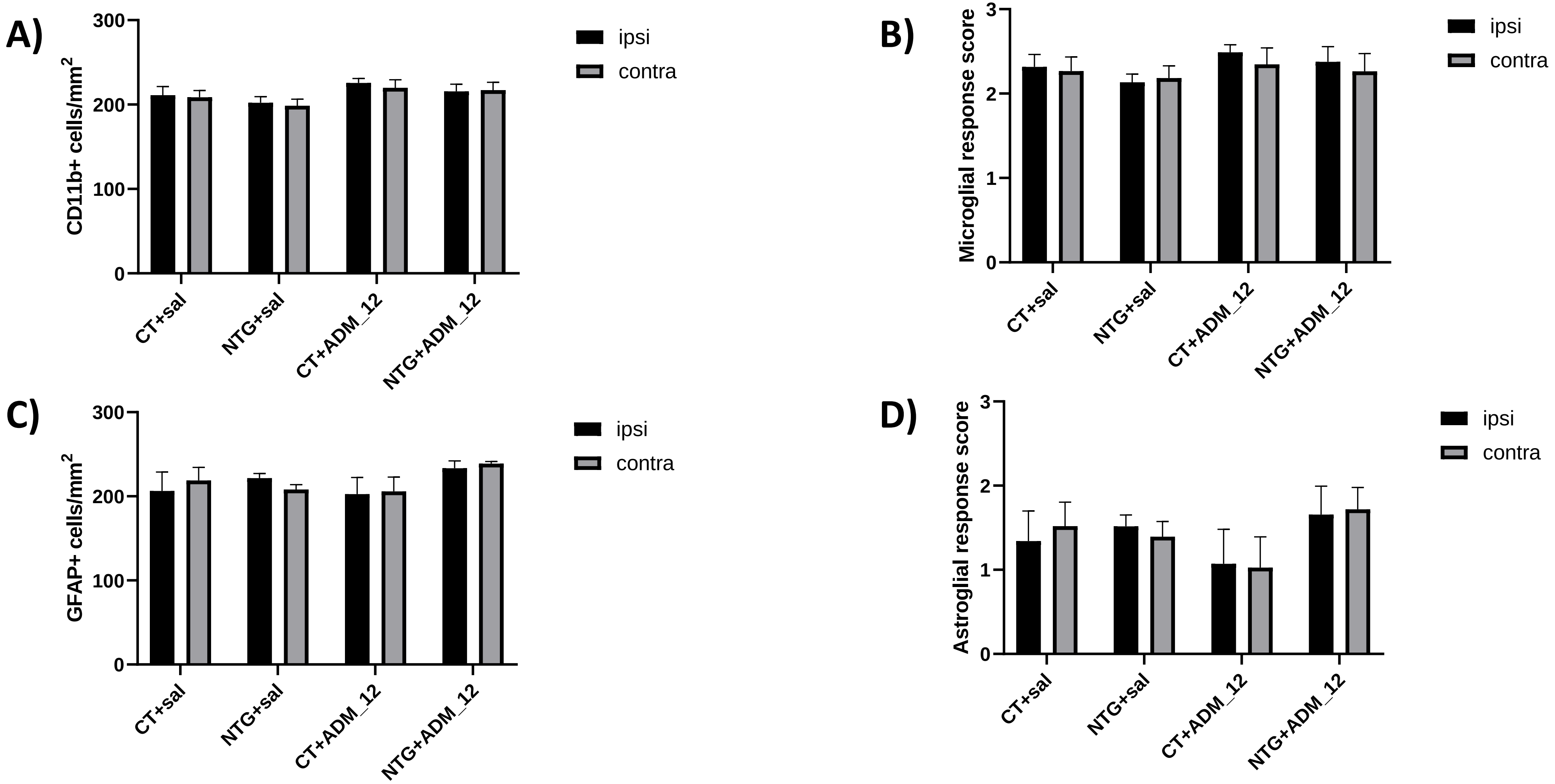

Figure S6. Chronic NTG model: comparison of immunofluorescence analysis data ipsi- and contralateral (with respect to formalin injection) TNC. Immunofluorescence analysis of the CD11b-positive cells per area in mm2 (**A**) and microglial response score (**B**). Immunofluorescence analysis of the GFAP-positive cells per area in mm^2 (**C**) and astroglial response score (**D**). Data are expressed as mean ± SEM. No statistically significant differences have been detected. Two-way ANOVA followed by Sidak's multiple comparisons test.

Table S1. Primer sequences obtained by the AutoPrime software (http://www.autoprime.de/AutoPrimeWeb)

Gene	Forward primer	Reverse primer
GAPDH	AACCTGCCAAGTATGATGAC	GGAGTTGCTGTTGAAGTCA
TNF-alpha	CCTCACACTCAGATCATCTTCTC	CGCTTGGTGGTTTGCTAC
IL-1beta	CTTCCTTGTGCAAGTGTCTG	CAGGTCATTCTCCTCACTGTC
IL-6	TTCTCTCCGCAAGAGACTTC	GGTCTGTTGTGGGTGGTATC
IL-10	GCTCAGCACTGCTATGTTGC	CAGTAGATGCCGGGTGGTTC
iNOS	TGGCCTCCCTCTGGAAAGA	GGTGGTCCATGATGGTCACAT
GFAP	GATGTAGGAGTGGGTAGGGC	CCCTCTCCGCATCCATACTT
CGRP	CAGTCTCAGCTCCAAGTCATC	TTCCAAGGTTGACCTCAAAG
